# Use of a Community Center Primary Care Clinic and Subsequent Emergency Department Visits Among Unhoused Women

**DOI:** 10.1001/jamanetworkopen.2021.3134

**Published:** 2021-03-25

**Authors:** Jenell Stewart, Kathryn M. Stadeli, Kristjana H. Ásbjörnsdóttir, Margaret L. Green, Giana H. Davidson, Bryan J. Weiner, Shireesha Dhanireddy

**Affiliations:** 1 Department of Global Health, University of Washington, Seattle; 2 Division of Allergy and Infectious Diseases, Department of Medicine, University of Washington, Seattle; 3 Department of Surgery, University of Washington, Seattle; 4 Department of Epidemiology, University of Washington, Seattle

## Abstract

This cohort study evaluates the association between use of a community center primary care clinic and subsequent nonemergent emergency department (ED) visits by unhoused women who exchange sex and inject drugs.

## Introduction

In the United States, the frequent amalgamation of homelessness, drug use, and exchange of sex for money, shelter, food, or drugs increases the risk of serious health conditions, including trauma and HIV.^1^ Women who are unhoused have an all-cause mortality 5 to 8 times higher than housed women.^2^ Seattle, Washington, is facing a housing crisis, with a 31% increase in homelessness from 2008 to 2018^3^ and a growing epidemic of opioid and methamphetamine use. In 2018, these overlapping epidemics created the context for an HIV outbreak in Seattle among unhoused heterosexual people.^4^

Several US cities have created mobile health clinics in an effort to engage unhoused people in medical care.^5^ In July 2018, the Safe, Healthy, Empowered (SHE) Clinic was founded to provide care for unhoused women colocated at Aurora Commons, a drop-in community center with a long-standing relationship with unhoused people in Seattle. Patients at the SHE Clinic reflect the unhoused population’s high rates of injection drug use, exchange sex, unplanned pregnancy, and sexually transmitted infections.^6^ In this cohort study, we evaluated the association between use of the SHE Clinic and the frequency of nonemergent emergency department (ED) visits among women living unhoused who exchange sex and inject drugs.

## Methods

Survey data collected from an observational cohort of 76 women 18 years or older at Aurora Commons included self-identified demographic characteristics, fertility desires, and HIV risk factors. Participants provided oral and written consent for surveys and abstraction of electronic medical record data. REDCap (Research Electronic Data Capture software (Vanderbilt University) was used for survey collection and consenting participants. This study received ethical approval from the University of Washington Institutional Review Board. The study followed the Strengthening the Reporting of Observational Studies in Epidemiology (STROBE) reporting guideline.

The SHE Clinic operates 4 hours a week and provides walk-in care. Participants with at least 1 visit to the SHE Clinic before February 2, 2019, were compared with participants with no visits. A manual review of participants’ electronic medical records from February 1, 2018, through August 1, 2019, was conducted to find the dates and chief concerns for ED visits in the Seattle area (10 facilities, 91% of the area EDs). Two clinician investigators (J.S. and M.L.G.) classified each ED visit as emergent or nonemergent. Emergency department visits for sexual assault, trauma, drug overdose, mental health crisis, or illness resulting in hospitalization were classified as emergent visits. Descriptive data analyses were performed with Stata version 15.0 software (StataCorp). Emergency department visit rates were calculated per woman per month, and paired Wilcoxon signed rank tests were used to compare the frequency of ED visits in the 6 months before and after a woman’s first SHE Clinic visit or a midperiod reference date for nonadopters.

## Results

A total of 76 cisgender women completed surveys. The median age of respondents was 37 years (interquartile range, 30-43 years). Forty-four women (58%) reported injection drug use in the past 3 months. Fifty-one women (67%) reported prior sexually transmitted infections. Forty-six women (60%) were tested for both *Chlamydia trachomatis* and *Neisseria gonorrhoeae,* and 40 women (53%) were tested for *Trichomonas vaginalis*; of these, 6 women (15%) had positive test results for *C trachomatis*, 5 (11%) for *N gonorrhoeae*, and 19 (48%) for *T vaginalis*. Four SHE Clinic patients were infected with HIV. Eligibility for HIV pre-exposure prophylaxis among women without HIV was high—76% (n = 55)—due to participation in transactional sex (n = 41), diagnosis of sexually transmitted infections (n = 25), and use of injection drugs (n = 44). Most women (67 [88%]) reported actively not wanting to become pregnant. Of the 48 women who were currently trying to avoid pregnancy (excluding 12 women with known infertility), only 8 women (17%) used contraceptives (Table). Forty-one women (54%) had established care at the SHE clinic.

**Table.** Self-reported Demographic Characteristics, HIV Risk Behaviors, and Fertility Desires Among 76 Women Accessing Day-Shelter Services in Seattle by Use of a Colocated Clinic

Characteristics	SHE Clinic users, No. (%)		*P* value
	Early adopter (n = 41)	Nonadopter (n = 35)	
Age, median (IQR), y	37 (30-43)	39 (32-45)	.41
Racial/ethnic identity			
White	22 (54)	16 (46)	.45
Black	5 (12)	4 (11)	
Native American/Alaska Native	4 (10)	8 (23)	
Black, biracial other	4 (10)	5 (14)	
Asian	1 (2)	1 (3)	
Other^a^	5 (12)	1 (3)	
Latinx identifying	3 (7)	6 (17)	.19
Currently unhoused	36 (88)	29 (83)	.54
History of injection drug use	30 (73)	23 (66)	.60
Injection drug use in past 3 mo	26 (63)	18 (51)	.42
Heroin	24 (59)	15 (43)	.43
Methamphetamine	23 (56)	17 (49)	.50
Transactional sex in past 3 mo	23 (56)	11 (31)	.04
Multiple sexual partners in past week	17 (41)	8 (23)	.69
No current pregnancy desire	36 (88)	31 (89)	.82
Surgically sterile	7 (17)	5 (14)	.78
Currently using contraceptives (pills, IUD, implant, or injection methods)	4 (10)	4 (11)	.78
Self-reported history of STI or vaginitis	35 (85)	22 (63)	.02
Bacterial vaginosis	22 (54)	14 (40)	.20
Trichomoniasis	21 (51)	8 (23)	.01
* Chlamydia*	25 (61)	15 (43)	.09
Gonorrhea	16 (39)	9 (28)	.19
Syphilis	2 (5)	1 (3)	.65
HIV positive	2 (5)	2 (6)	.87
Hepatitis C antibody positive	19 (46)	9 (26)	.08

Abbreviations: IQR, interquartile range; IUD, intrauterine device; SHE, Safe, Healthy, Empowered; STI, sexually transmitted infection.

^a^ The other racial/ethnic identity option was selected when participants did not believe that their personal identity could be captured by census categories.

Most women (67 [88%]) visited an ED during the 18-month study period and commonly had more than 3 ED visits (46 [61%]). Of the 388 ED visits by respondents, 168 (43%) were for emergent concerns, and 220 (57%) were for nonemergent concerns, such as isolated infections or a chronic condition. The frequency of visits for nonemergent concerns decreased among SHE Clinic patients from 37 visits in the 6-month pre-intervention period (mean, 0.50 visits per 100 woman-days; 95% CI, 0.32-0.68 visits per 100 woman-days) to 22 visits in the 6 months thereafter (mean, 0.30 visits per 100 woman-days; 95% CI, 0.15-0.45 visits per 100 woman-days) (*P* = .02). No change in the frequency of nonemergent visits was observed among nonadopters: 37 visits in the pre-intervention period (mean, 0.59 visits per 100 woman-days; 95% CI, 0.33-0.85 visits per 100 woman-days) vs 38 visits after the intervention (mean, 0.60 visits per 100 woman-days; 95% CI, 0.28-0.92 visits per 100 woman-days) (*P* = .51).

## Discussion

This was an observational study of real-life medical care use and has some limitations, including room for sampling bias because of the small sample size. Nevertheless, this study’s findings suggest that use of colocated clinic services may be associated with reduced nonemergent ED visits among unhoused women in Seattle, indicating the need for walk-in harm-reduction services.
